# Development and Psychometric Properties of the Synthetic Drug Dependence Scale in a Chinese Sample

**DOI:** 10.3389/fpsyg.2021.717029

**Published:** 2021-10-26

**Authors:** Mei-Ting Li, Jun Zhang, Dong-Cheng Zhang, Qing-Qing Che, Ze-Lan Liu, Pei-Wen Yang, Xin-Wei Luo, Tai-Sheng Cai

**Affiliations:** ^1^The Medical Psychological Institute, Second Xiangya Hospital, Central South University, Changsha, China; ^2^Department of Corrective Education, Hunan Judicial Police Vocational College, Changsha, China; ^3^School of Education, South-Central University for Nationalities, Wuhan, China

**Keywords:** synthetic drugs, dependence, measurement invariance, psychometrics, traditional drugs

## Abstract

**Objective:** In contrast to the drug situation in the rest of the world, synthetic drugs, rather than traditional drugs, have been the dominant abused drugs in China since 2019. However, the public misconception that synthetic drugs are not as addictive as traditional drugs, such as opioids and the scarcity of specific measurement instruments, have hindered the clinical diagnosis and treatment of synthetic drug abusers, thus the development of a localized instrument to evaluate dependence on synthetic drugs is in urgently needed.

**Method:** Using a sample of 618 Chinese synthetic drug abusers (Mean age = 34.69 years; 44.17% female), the present study developed and examined the psychometric properties of a self-reporting instrument, the Synthetic Drug Dependence Scale (SDDS), which consists of four subscales: physical dependence, psychological dependence, health injury, and social function injury.

**Results:** The SDDS revealed a three-factor model structure (weighted root mean square residual (WRMR) = 0.876, comparative fit index (CFI) = 0.965, Tucker–Lewis index (TLI) = 0.953, and Root mean square error of approximation (RMSEA) = 0.070), with good internal consistency (composite reliability = 0.912, alfa = 0.801) and convergent validity. Elevated scores on the SDDS were associated with a higher level of reward sensitivity, punishment sensitivity, and stronger impulsivity. Interestingly, psychological dependence was the only significant predictor (*p* < 0.05) of criterion variables compared with the other three subscales, implying the important role of psychological factors in synthetic drugs dependence. Adequate measurement equivalence across sex, age (18–30 and 31–57 years old), and employment group (employed and unemployed) was also established.

**Conclusion:** The SDDS appears to be an effective and reliable instrument that could be used to further investigate the characteristics of synthetic and traditional drug dependence, promoting a deeper understanding of the physical and psychological roles in drug dependence.

## Introduction

Synthetic drugs, compared with traditional drugs derived from natural plants, are artificially synthesized from chemical substances (Sacco and Finklea, 2012), accompanied by alarming health and society problems. Synthetic drug abuse leads to neurological changes, such as white matter abnormalities, cognitive impairment, (Morgan and Curran, 2012), and psychotic symptoms, such as delusions and hallucinations (Stoica and Felthous, 2013). Besides, stimulants make people overexcited or hyperactive, and they are more likely to exhibit violent behavior and even commit serious criminal acts (Zweben et al., 2004), posing a huge threat to public security. It is worthy to note that drug abuse is a global challenge that differs in terms of its performance and characteristics among different countries and regions (Kumar and Mittal, 2020). As of 2018, the global number of past-year synthetic drug users has grown to over 48 million (United Nations Office on Drugs and Crime, 2020), however, until now, most studies have focused on drug problems in Western countries using research models designed for Western countries, despite these countries representing only a small fraction of the population of the world (West et al., 2019). In stark contrast, faced with language barriers and resource constraints, non-western studies have received little attention and interpretation, let alone the further application and amelioration, which may finally come to a standstill.

With 18% of the population of the world and 16% of global gross domestic product (The world bank, 2019), it is essential for China to construct localized research and develop measurement tools to determine the features of synthetic drug dependence in Chinese culture, which might promote a deeper understanding of drug problems in the world. Currently, synthetic drugs have replaced traditional drugs as the most abused drugs in China. From 2004 to 2019, the proportion of synthetic drugs abusers increased from 9.5 to 57.5% (Office of China National Narcotics Control Commission, 2005, 2020). Unfortunately, current measurement tools, which mainly focus on traditional drugs, have failed to keep pace with the changing situation. Without a targeted evaluation instrument, it would be difficult to carry out preliminary screening at the population level, evaluate treatment needs, and develop personalized rehabilitation programs, thus holding back the clinical diagnosis and treatment of synthetic drug dependence.

Another challenge is the illusion among many people that synthetic drugs are less addictive than traditional drugs, and it is thus “safe” to take synthetic drugs (Zeng et al., 2016). This lack of knowledge partly results from the fact that the physical symptoms associated with synthetic drugs are not as obvious as those of traditional drugs, for example, withdrawal symptoms are common in traditional drugs dependence, whereas they have not been documented for some synthetic drugs, such as ketamine, phencyclidine, and other hallucinogens (Wang et al., 2010; American Psychiatric, 2013). However, the psychological dependence potential might be stronger, in that it is faster for synthetic drug abusers to develop from trial use to compulsive use (Liu, 2005). Notably, such a difference does not indicate that one is more addictive than the other, nor that one is safer than another, which warrants the development of an objective index to distinguish and compare the specific characteristics between traditional drugs and synthetic drugs, serving to grasp the nature of drug dependence.

Based on the above discussion, current research focuses on two relationships, one is the relationship between China and world drug problems, second is the relationship between traditional drugs and synthetic drugs. For the first one, compared with the tendency for drug legalization in many western countries, the Chinese government has always adopted a stricter and cautious attitude to drug administration, thus a localized instrument would be more suitable for Chinese drug users. As for the second one, it is inappropriate to define synthetic drug dependence directly using the characteristic of traditional drugs because of their difference, nor should we completely separate synthetic drugs from traditional drugs because of their similarity. A better choice is to develop a universal scale to avoid inconsistent components and focus on the common elements and fundamental characteristics of drugs dependence. Therefore, the present study aimed to adapt the Opiate Addiction Severity Inventory (OASI), which is widely used for Chinese opiate abusers, for application in the dependence rating of synthetic drugs.

The OASI is a self-report instrument used to measure the severity of opiate dependence, with good reliability and validity for both the community-based drug users and rehabilitation drug users (Lian and Liu, 2004; Gu et al., 2008). It consists of four subscales: physical dependence, psychological dependence, health injury, and social function injury. Physical dependence is not the tolerance or withdrawal symptoms different for various drugs but is the objective frequency and dosage of drug use. Psychological dependence includes the craving for a drug, attempts to quit drugs, and time spent on drugs. Health injury and social function injury reflect the health and social problems caused by drug use. Except for a few items referring to the specific characteristics of opiate dependence, the remaining items contain parts shared by synthetic drugs and traditional drugs; therefore, it is feasible to revise items to develop a measure with wider application, akin to the adaptation of the Questionnaire on Smoking Urges to Marijuana Craving Questionnaire (Heishman et al., 2001) and the Alcohol Craving Experience to the Craving Experience Questionnaire (May et al., 2014). Besides, the four subscales measure various important aspects of dependence that can provide a more comprehensive assessment of synthetic drug dependence compared with a unidimensional scale, such as the Severity Dependence Scale (Gossop et al., 1995). Thirdly, compared with interview instruments, such as the Diagnostic and Statistical Manual of Mental Disorders and the Addiction Severity Index, the OASI only has 12 items and is thus more convenient to perform population-based assessments with a lower time cost (Baggio et al., 2020; Yerima et al., 2020), which is conducive to establish prevalence rates at the population level. Furthermore, as a Chinese localized scale, the content of the scale better fits with the Chinese social and cultural background (Lian and Liu, 2003, 2004) and containing natural and intelligible item wording without distorting part of the information during translation (Upsing and Rittberger, 2018), thus the scale will be more suitable for Chinese drug abusers and will produce more authentic information compared with directly applying western models (West et al., 2019).

The following two steps were performed to adapt the OASI for application in the dependence rating of synthetic drugs. The first step was to develop a Synthetic Drug Dependence Scale (SDDS) and then examine the psychometric properties of the scale, consisting of the following procedures: (a) item revision. Several items are specific for opioid abusers needed to be modified for their generalizing to synthetic drugs. (b) Factor structure confirmation. (c) Internal consistency and convergent validity evaluation. Item reliability, composite reliability, and Cronbach's alfa jointly acted as indicators of internal consistency, and drug craving, reinforcement sensitivity, and impulsivity were chosen as the validation variables to evaluate the convergent validity. Reinforcement sensitivity and impulsivity are closely associated personality traits with drug abuse. Those with high reward sensitivity are more attentive to the euphoric effects of drug taking (O'Connor et al., 2009) which raises the risk for drug abuse (Papinczak et al., 2018). Findings have proved that those with strong punishment sensitivity may deal with the aversive stimulation in a maladaptive way (Le et al., 2019), such as alcohol drinking (Kuntsche et al., 2008) or drug taking, thus increasing the dangers of drug abuse (Voigt et al., 2009). Besides, the impulsive individuals have a heightened propensity (Gullo et al., 2014) to take drugs with a lessened ability to control drug use (Zeng et al., 2016), which finally leads to more drug-related problems and dependence (Wardell et al., 2016).

The second step was to assess the measurement invariance of the scale so as to make comparisons between groups valid (Deng et al., 2008; Putnick and Bornstein, 2016). Studies have shown that females are more sensitive than males to physical and psychological effects of drugs (Anker and Carroll, 2011; Simmler et al., 2011) and present more severe negative drug-related problems related to ecstasy (Allott and Redman, 2007), ketamine (Li et al., 2017), methamphetamine (Kogachi et al., 2017), and other substance use (Fattore et al., 2009; van der Plas et al., 2009), indicating the importance of understanding sex differences to better guide drug investigation and clinical treatment. In Belarus, compared with people over 30 years old, young drug users under 30 years old had a higher prevalence of the use of hallucinogens and amphetamine-type stimulants (Lelevich et al., 2016), and the rates of substance use disorder are highest in individuals younger than 30 years, for both the cocaine users and hallucinogen users (American Psychiatric, 2013). Besides, illicit drugs are expensive, thus socioeconomic factors are germane to drug abuse, affecting the accessibility to drugs. Unemployment, as a negative life event, would result in emotional stress (Catalano et al., 2011) and lower life quality (Burgard et al., 2013), which might increase the risk of drug use and the subsequent development of drug dependence (Henkel, 2011). People who have spent a long time unemployed report more drug abuse (Glei and Weinstein, 2019), signifying the potential implications of vocational promotion for drug rehabilitation. Therefore, the current study assessed the measurement equivalence between males and females, young people (18–30 years old), middle-aged people (31–57 years old), and employed and unemployed people to more accurately compare sociodemographic differences.

## Methods

### Participants and Procedures

The survey was administered in four compulsory drug rehabilitation centers of Hunan province, China, between July and August 2019. There were 176,000 drug users in Hunan province, ranking third in China, and accounting for 7.32% of the total number of drug users. Eligibility criteria required that a participant was at least 18 years old, mainly took synthetic drugs before entering the rehabilitation center, and was willing to participate in the survey. Participants were excluded if they did not want to join in or were accompanied by serious physical diseases, mental deficiency, language disorders, or any other problem that could interfere with their understanding of the questionnaire. During the testing process, correctional staff was responsible for organizing participants to enter and exit the test site and was not allowed to touch any of the survey materials. They were also asked to avoid watching subjects while they answered the questionnaire and to remain a far distance from them. Besides, participants were spaced from each other to prevent discussion and ensure privacy. Then, the participants were told that the questionnaires would help to improve their treatment plan and there were no trick questions, and no right or wrong answers. More importantly, their results would not act as indicators for reward or punishment later, so they need not worry about the results. After that, researchers read and explained the survey questions and response options one by one, to make sure all the participants understood the questionnaire, reading glasses were handed out to those in need.

Overall, 650 participants joined in the survey and 618 valid SDDS questionnaires were left after deleting the incomplete and problematic questionnaires, such as those with identical responses for all items. In terms of the demographic details, the mean age of the sample was 34.69 years (SD = 7.29; range: 18–57 years) and 44.17% were female (*n* = 273). The education level of most people was under high school (66.18%); 369 had jobs (59.71%) and 240 were unemployed (38.83%). As for their marital status, 187 (30.26%) were single, 149 (24.11%) were married, and 198 (32.04%) had a broken relationship, such as separation or divorce. Among them, 374 participants also completed the correlated validity scales and 370 validity samples were gained. Lastly, 100 participants were chosen at random for retesting 1 month later. Ethical approval was obtained from the Institutional Review Board of the Second Xiangya Hospital of Central South University.

### Measures

#### SDDS

As introduced above, the SDDS is a 12-item self-reporting measure modified from the OASI scale to assess the severity of synthetic drug dependence, such as the frequency of drug use, time spent on the drug, and degree of impaired physical and social functioning. Respondents rated each item on a four-point scale and higher scores represent a greater degree of dependence.

The 48-item Sensitivity to Punishment and Sensitivity to Reward Questionnaire (SPSRQ) (Torrubia et al., 2001) measures the extent of behavior tendency toward punishment and reward with two subscales: sensitivity to punishment (SP) and sensitivity to reward (SR). Participants responded either “yes” or “no” to the item; a response of “yes” was assigned a value of one and “no” was assigned as zero. Items were summed separately to the total subscale scores and higher scores indicated stronger SP or SR. The SPSRQ demonstrated favorable internal consistency in the current study, with α = 0.83 (SP) and α = 0.81 (SR).

The 20-item Short Version of the UPPS-P Impulsive Behavior Scale (SUPPS-P) (Lynam, 2013) measures the degree of behavior impulsiveness from the following aspects: positive urgency, negative urgency, lack of perseverance, lack of premeditation, and sensation seeking. Respondents rated each item on a four-point scale from 1 (not at all true of me) to 4 (very true of me). Then, the items were summed to a total score reflecting overall impulsiveness, with higher scores indicating a stronger degree of impulsiveness. The S-UPPS-P evidenced adequate internal consistency (α = 0.77) in the current study.

### Data Analytic Strategy

#### Item Revision

The item revision was completed by the expert group consisted of five professors working within the psychometrics and drug dependence fields. Three experts revised the item wording and expression, and the remaining experts evaluated the content validity of all the items. Based on the OASI scale, 10 items kept the same wording except for the deletion of “heroin.” Besides, given the difference in specific behavior characteristics between traditional drugs and synthetic drugs, the two remaining items were refined to make them more suitable for synthetic drug users in the expert group meeting. The second item “amount of daily heroin use” was transformed to “amount of daily synthetic drugs use,” and the dosage range varied in methamphetamine, ketamine, and ecstasy according to their dosage features. The choice of these three synthetic drugs lies in the fact that they are the top three abused synthetic drugs in China (Bao et al., 2015; Office of China National Narcotics Control Commission, 2020) and are commonly used in current samples. The third item “interval between waking up and first heroin taking” was modified to “as the duration of drug use increases, your desire for drugs becomes stronger” because there is no such behavior feature for synthetic drug users to take drugs after getting up, which is an important behavior symptom of opiate users (Liu et al., 2000). Even so, the modified third item was designed to measure the degree of drug craving over time, similar to the original item. The content validity of all items was established in the light of representativeness, comprehension, and clarity properties. Then, the SDDS was formed for application in subsequent investigations.

#### Item Analyses

Item analyses were performed to check the psychometric property of every single item to make a decision regarding item modification or item deletion. First, means and variance of items were displayed to calculate and compare the coefficient of variation (C.V.), for which a low value indicated higher stability and less variability of data. Then skewness and kurtosis were inspected to examine the distributional properties that influence the choice of subsequent estimation methods (Gao et al., 2008). Given the fact that it would not be reasonable to expect the observed data to conform to a standard normal distribution in the drug use or psychopathology area (Curran et al., 1996). A moderately non-normality distribution was hypothesized and the acceptable guideline was 2 for univariate skewness, 7 for univariate kurtosis (Finney and DiStefano, 2006), and 7.98 for multivariate kurtosis (Gao et al., 2008). In addition, group discrimination analyses were used to assess the extent to which each SDDS item distinguishes between high- and low-synthetic drug dependence groups. Subjects were put in the high group if they scored in the top 72% on the SDDS score (*n* = 180, mean = 23.170, SD = 2.730) and were placed in the low group if they scored in the bottom 27% on the SDDS score (*n* = 195, mean = 9.833, SD = 2.618). Lastly, internal consistency was evaluated using the corrected item-total correlation, and a value higher than 0.30 was acceptable (Nunnally, 1994). An item should be removed if it fails to discriminate between the high and low groups or the value of the corrected item-total correlation is under 0.30.

#### Factorial Structure

To determine the final model structure and assess the construct validity of the SDDS, the data were divided randomly into two samples, training sample contained 326 participants for exploratory factor analysis (EFA), and the validation sample had 292 participants for confirmatory factor analysis (CFA). SPSS 19.0 (IBM Corp, 2010) was used for the EFA. Given that the ML-estimated EFA model can be distorted when the data depart from a multivariate normal distribution (Costello and Osborne, 2005), the principal axis factor estimation which is free of distributional assumptions (Brown, 2015) was chosen as the factor extraction method. Scree plot and oblique rotation were performed as the correlations between factors were expected. Then the CFA was conducted to examine the factor structure demonstrated by the results of EFA.

#### Factorial Validity

All models were conducted in Mplus7.0 (Muthén and Muthén, 1998). Weighted least squares mean and variance-adjusted estimator (WLSMV) was applied as it is more suitable for ordered categorical items with five or fewer response options (Bandalos, 2014). Besides, when the sample size is <500 (the validity sample size was 370), the generalized least square (GLS) method can provide more tenable estimation results (Hu et al., 1992); therefore, the GLS estimation was applied in the statistical analysis of the validity sample.

Two indices were used to evaluate the model fit: the preliminary fit criteria and the overall model fit (Bagozzi and Yi, 1988). The preliminary fit criteria aimed to detect the existence of model specification errors or identification problems. The most common anomalies are negative error variance, correlation >1, and extreme parameter estimates. Then, the overall model fit was used to examine the similarity between the theoretical model and the sample data, such as the absolute fit, comparative fit, and parsimony fit. The indices of absolute fit are χ^2^ and weighted root mean square residual (WRMR) which was developed for ordinal data when categorical data are analyzed (Muthén, 2004), the smaller their values are, the better the model fits the sample data. However, the χ^2^ will inflate by sample size, hence a statistically significant χ^2^ could not prove that a model fits the data poorly (Brown, 2015). Comparative fit evaluates the distinction between the theoretical model and the null model, the bigger the difference is, the better the model fits the data accordingly. The common indices are the comparative fit index (CFI) and the Tucker–Lewis index (TLI). Parsimony fit takes the model simplicity into account when choosing from two models fitting the data sample equally well at absolute and comparative levels. Root mean square error of approximation (RMSEA) is one of the parsimony fit indices for which a smaller value indicates a more parsimonious model. The following guidelines were used: WRMR should be lower than 1.0 (DiStefano et al., 2017), CFI and TLI should be near 0.95 (Hu and Bentler, 1999), RMSEA should be less than 0.06, and the upper limit of 90% RMSEA CI should not exceed 0.10 (Kline, 2015).

#### Internal Reliability and Convergent Validity

It is crucial to scrutinize the internal structure of the scale after determining the final factor structure. Item reliability, composite reliability, and Cronbach's alfa are normally tested. Item reliability (the square of standardized factor loading) should be lower than the composite reliability, whose values >0.60 are desirable (Bagozzi and Yi, 1988). The common guideline of Cronbach's alfa is 0.70 (Nunnally, 1994); however, values between 0.60 and 0.70 are acceptable and under 0.60 is possible when the factor consists of a few items (Krank et al., 2011). Next, test-retest reliability was examined by correlating factor scores of the same subjects at an interval of 1 month.

Convergent validity was then tested by correlating the subscales with external variables in the validity sample. Guidelines for thresholds of small (*r* = 0.10), medium (*r* = 0.30), and large effect size (*r* = 0.50) were adopted (Cohen, 1992). The reinforcement sensitivity and impulsiveness act as behavior tendencies affected by complex external factors; therefore, the correlations were expected to be positive with small-to-medium size. Based on the results of the correlation analysis, structural regression, in which the factors act as predictors of external variables, was then performed to further investigate the predictive power and separative role of the subscales.

#### Measurement Invariance

A multigroup confirmatory factor analysis (MCFA) was conducted separately to test measurement invariance between males and females, young and middle age, and employed and unemployed people in the final model. Testing measurement invariance consists of three procedures from the least restrictive level to the most restrictive level: (a) configural invariance (i.e., equal structural form) is the base model that must be established to make subsequent tests meaningful. (b) Metric invariance (i.e., equal factor loadings) is the prerequisite for unambiguous cross-group comparison (Bollen, 1989). (c) Scalar invariance, also called threshold invariance for ordinal data, implies that members of both groups have an equal probability between response categories (e.g., strongly disagree to disagree), making scores on latent factor comparable (Kim and Yoon, 2011).

For the invariance indices, a non-significant χ^2^ difference between models is the normal index and the Satorra–Bentler chi-square (ΔSB-χ^2^) can be used in place of the standard Δχ^2^ value in analyses of ordinal data (Asparouhov et al., 2006); however, it could easily result in over-rejection of invariant models as the sensitivity to sample size (Kline, 2015). Alternative indices are used in conjunction with Δχ^2^ to evaluate model fit, i.e., changes in CFI ≤ 0.01 (Cheung and Rensvold, 2002), changes in TLI ≤ 0.05 (Little, 1997), and an increase in RMSEA ≤ 0.015 (Chen, 2007) indicate that the null hypothesis of model invariance should not be rejected and an equivalent model is thus established.

## Results

### Item Analyses

Table 1 presents the analyses of the SDDS items. Using the coefficient of variation as an indicator of the degree of variation, values of all the items were under 1.0, except item 5. Besides, skewness and kurtosis of all the items ranged from −2 to 2, and the multivariate kurtosis was 10.886 for the scale, which demonstrated a moderate non-normal distribution. For the item discrimination analyses, significant group differences were observed for all items (*p* < 0.001, Hedges' g = 4.993) and participants in the high-synthetic drug dependence group scored significantly higher than those in the low-synthetic drug dependence group on the SDDS total score (*p* < 0.001). The internal consistency was evaluated using the corrected item-total correlation, and item 5 was identified as unacceptable based on the criterion of 0.30 (Nunnally, 1994). In view of the omnibus tests, item 5 needed to be deleted from the scale. After deletion, the multivariate kurtosis score decreased to 7.551, and the corrected item-total correlation was conducted again, which revealed that no other problematic item existed.

**Table 1:** Analysis of the 12 items of the synthetic drug dependence scale.

**Item**	**M**	**SD**	**C.V**.	**Skewness**	**Kurtosis**	**Discrimination-t**	**CITC**	**Item 5-deleted CITC**
1	1.259	0.958	0.761	0.291	−0.839	17.907	0.484	0.474
2	1.459	1.100	0.754	0.172	−1.247	14.025	0.353	0.355
3	1.641	0.947	0.577	−0.090	−0.928	20.046	0.529	0.532
4	1.757	0.853	0.485	−0.065	−0.784	16.615	0.515	0.519
5	0.405	0.582	1.437	1.318	1.757	5.468	0.146	–
6	1.154	0.803	0.696	0.215	−0.529	18.130	0.542	0.532
7	1.477	0.723	0.490	0.208	−0.246	14.214	0.451	0.456
8	0.917	0.807	0.880	0.356	−0.859	11.508	0.348	0.339
9	1.426	0.942	0.661	0.040	−0.900	17.881	0.533	0.540
10	2.083	0.796	0.382	−0.655	0.072	14.293	0.443	0.455
11	1.275	0.708	0.555	0.927	0.836	13.203	0.431	0.435
12	1.521	0.949	0.624	0.014	−0.917	17.980	0.476	0.477

*C.V., coefficient of variation; CITC, corrected item-total correlation*.

### Factorial Structure

In the first step of the analysis, the preliminary fit was tested for the three measurement models. It turned out that no negative error variance or correlation >1 existed, and all the parameter estimates were in the normal range, laying the foundation for subsequent examination. As for the results of EFA, the Kaiser–Meyer–Olkin measure of sampling adequacy (0.843) and Bartlett's Test of Sphericity (*p* < 0.001) indicated the adequacy of the current sample for factor analysis. This initial analysis produced a two-factor structure accounting for 47.33% of the observed total variance. Given the four-factor model proposed by the original scale author (Lian and Liu, 2004), the EFA was run again by fixing four factors to extract. However, as was shown in Table 2, A1, A9, A10, and A11 did not have the highest loadings in their theoretically corresponding factor, which failed to support the four-factor model. Based on the above results of two-factor structure, four-factor structure and the original author's study of combing the physical injury factor and social function injury factor into the functional impact factor (Gu et al., 2008), the current study attempted to establish the three-factor model, which is to retain the physical dependence factor and psychological dependence factor, and merge the physical injury factor with the social function injury factor into the functional impact factor. Therefore, the EFA has been performed again by fixing three factors to extract, it produced a three-factor structure accounting for 55.45% of the observed total variance and the factor loadings of each item conformed to the three-factor structure. Then, the two-factor model and three-factor model were compared in the subsequent CFA analysis.

**Table 2:** Factor loadings from factor analyses in samples 1 and 2.

	**Sample 1**									**Sample 2**				
	**EFA 1**		**EFA 2**				**EFA 3**			**CFA 1**		**CFA2**		
**Item**	**1**	**2**	**1**	**2**	**3**	**4**	**1**	**2**	**3**	**1**	**2**	**1**	**2**	**3**
1	**0.613**	0.299	0.543	**0.564**	0.324	−0.109	**0.682**	0.517	0.291	0.676		0.803		
2	**0.467**	0.155	**0.700**	0.404	0.169	−0.075	**0.551**	0.367	0.146	0.546		0.624		
3	**0.712**	0.366	0.375	**0.752**	0.355	0.016	0.490	**0.727**	0.336	0.766			0.768	
4	**0.688**	0.349	0.416	**0.689**	0.326	0.124	0.471	**0.706**	0.319	0.724			0.726	
5	**0.571**	0.397	0.309	**0.581**	0.381	0.124	0.388	**0.578**	0.377	0.763			0.766	
6	0.343	**0.574**	0.151	0.369	**0.580**	0.110	0.231	0.354	**0.574**		0.587			0.587
7	0.177	**0.487**	0.101	0.190	**0.494**	0.134	0.122	0.184	**0.498**		0.494			0.494
8	0.377	**0.637**	0.103	0.433	**0.624**	0.554	0.122	0.465	**0.634**		0.773			0.773
9	0.216	**0.660**	0.076	0.249	**0.654**	0.229	0.101	0.254	**0.667**		0.739			0.739
10	0.377	**0.493**	0.181	0.409	**0.538**	−0.082	0.286	0.370	**0.491**		0.627			0.626
11	0.335	**0.573**	0.075	0.385	**0.553**	0.235	0.123	0.404	**0.567**		0.732			0.733

*EFA, exploratory factor analysis; CFA, confirmatory factor analysis. Values in bold indicate the factor on which the item is loaded*.

### Factorial Validity

Results of CFA indicated that two-factor structure yielded good fit to the data: χ2/df = 107.024/43, WRMR = 0.928, CFI = 0.961, TLI = 0.951, and RMSEA = 0.071 [0.055, 0.089]. Compared with the two-factor model, the three-factor model evidenced superior fit to the data: χ2/df = 98.943/41, WRMR = 0.876, CFI = 0.965, TLI = 0.953, and RMSEA = 0.070 [0.052, 0.087], with higher factor loadings of items which demonstrated a stronger explanatory power of the factors. Combined with the low overall variance explanation rate of the two-factor structure in the EFA analysis, three-factor model was determined as the final factor structure of the SDDS.

It is worth noting that the factor loading of item 7 is 0.494, which may be related to the cultural characteristics reflected in the item. Item 7 asked about the changes in sexual behaviors and desire, 74.9% of the answers of the subjects focused on the first two options, indicating that most participants in the current sample thought that drug taking had no effect or a slightly negative effect on their sexual lives. In Chinese culture, however, the attitude of people toward sex is relatively conservative, and sex-related topics are rarely being talked (Higgins et al., 2002), so that they would be ashamed in admitting their lower sexual drive and more likely to underestimate the changes in sexual behavior, which finally leads to the positive skew distribution and lower factor loading of the item.

### Internal Reliability and Convergent Validity

As shown in Table 3, all items had significant loadings on the factors with standardized parameter estimates ranging from 0.50 to 0.82. In addition, the composite reliability of the factor was desirable, with values >0.65 and higher than its corresponding item reliability. Consistent with prediction, the Cronbach's alfa score of the physical dependence factor was lower than 0.60, because the factor included only two items and the Cronbach's alfa score of the other factors was >0.60. Besides, the test-retest correlations of the subscales and total scale ranged from 0.500 to 0.672, which could mainly be explained by the characteristics of the subjects. First, items in the physical dependence factor required subjects to recall the dosage and frequency of synthetic drug use, which the subjects found hard to recall in terms of the exact numbers, and the discrepancies seemed to be rooted in a legitimate difficulty with recall. Next, there was a tendency among the subjects to report a lower degree of dependence during the retest (McLellan et al., 1985), which might have resulted from the lack of drug-related clues and the strict management of the drug rehabilitation center. On the whole, these reliability estimates provided support for adequate internal reliability and retest reliability of the total scale and the subscales.

**Table 3:** Internal reliability and test-retest reliability of the SDDS.

**Scale**	**Item**	**Loading**	**IR**	**CR**	**Alfa**	**Test-retest reliability**
Physical dependence	1	0.803	0.645	0.678	0.584	0.500^**^
	2	0.624	0.389			
Psychological dependence	3	0.768	0.59	0.798	0.723	0.672^**^
	4	0.726	0.527			
	5	0.766	0.587			
Functional impact	6	0.587	0.345	0.824	0.752	0.603^**^
	7	0.494	0.244			
	8	0.773	0.598			
	9	0.739	0.546			
	10	0.626	0.392			
	11	0.733	0.537			
Total scale	–	–	–	0.912	0.801	0.656^**^

*IR, item reliability; CR, composite reliability; Alfa, Cronbach's alfa.*

** *p < 0.01*.

Correlations between the SDDS and related variables can be found in Table 4, which indicated that there were significant positive correlations between subscales and other variables, except for the physical dependence subscale, indicating the potential specific role of physiological factors. Besides, the subscales had moderate associations with reward sensitivity, punishment sensitivity, and impulsiveness, as expected, supporting the good convergent validity of the scale. In subsequent structure regression analyses, the psychological dependence factor was the only significant predictor (*p* < 0.05) of related variables, with standardized coefficient estimates from 0.149 (impulsiveness) to 0.259 (SR), implying the importance of psychological factors in synthetic drugs dependence.

**Table 4:** Correlation coefficients between the SDDS and other related variables.

**Scale**	**SP**	**SR**	**S-UPPS-P**
Physical dependence	0.075	0.013	0.086
Psychological dependence	0.209^**^	0.215^**^	0.158^*^
Functional impact	0.249^**^	0.209^**^	0.146^*^

*SP, sensitivity to punishment; SR, sensitivity to reward; S-UPPS-P, Short Version of the UPPS-P Impulsive Behavior Scale.*

* *p < 0.05.*

** *p < 0.01*.

### Measurement Invariance

The results of measurement invariance are shown in Table 5. The results suggested that the fit indices for each group were adequate to good, providing evidence of configural invariance and laying the foundation for subsequent analyses. In the sex group, the metric invariance model was successfully established with non-significant Δχ^2^ and subtle change in CFI, TLI, and RMSEA scores, but not for the scalar invariance model. Then partial invariance test was conducted by releasing the second threshold of item 2 to be freely estimated (van de Schoot et al., 2012), it turned out that the ΔSB-χ^2^ was significant (Δχ^2^ = 32.865, df = 18, *p* = 0.017) with a smaller change in CFI (−0.004) and TLI (0.002) and continued to release the third threshold of item 2, the ΔSB-χ^2^ was insignificant (Δχ^2^ = 23.687, df = 17, *p* = 0.128) with acceptable change in CFI (−0.002) and TLI (0.005), hence the partial invariance was established.

**Table 5:** Measurement invariance across sex, age, and employment.

	**Model**	**Model fit**				**Model comparison**			
		**χ^2^ (df)**	**CFI**	**TLI**	**RMSEA [90%CI]**	**Δχ^2^ (df)**	***p***	**ΔCFI**	**ΔTLI**
Gender	Male participants	82.486 (41g6 g6)	0.976	0.968	0.054 [0.037, 0.071]	–	–	–	–
	Female participants	93.577 (41)	0.962	0.949	0.069 [0.050. 0.087]	–	–	–	–
	Configural model	176.420 (82)	0.970	0.960	0.061 [0.049, 0.073]	–	–	–	–
	Metric model	182.896 (90)	0.971	0.964	0.058 [0.046, 0.070]	11.347 (8)	0.183	0.001	0.004
	Scalar model	230.852 (109)	0.961	0.961	0.060 [0.049, 0.071]	51.052 (19)	<0.001	−0.010	−0.003
Age	Young participants	74.805 (41)	0.963	0.951	0.066 [0.041, 0.089]	–	–	–	–
	Middle participants	130.238 (41)	0.960	0.946	0.072 [0.058, 0.086]	–	–	–	–
	Configural model	206.484 (82)	0.960	0.947	0.070 [0.059, 0.082]	–	–	–	–
	Metric model	210.595 (90)	0.961	0.953	0.066 [0.055, 0.078]	11.790 (8)	0.161	0.001	0.006
	Scalar model	229.304 (109)	0.962	0.961	0.060 [0.049, 0.071]	28.556 (19)	0.073	0.001	0.008
Employment	Unemployed participants	76.420 (41)	0.969	0.959	0.060 [0.039, 0.081]	–	–	–	–
	Employed participants	108.058 (41)	0.966	0.954	0.067 [0.051, 0.082]	–	–	–	–
	Configural model	183.465 (82)	0.967	0.956	0.064 [0.051, 0.076]	–	–	–	–
	Metric model	183.424 (90)	0.970	0.963	0.058 [0.046, 0.070]	7.206 (8)	0.515	0.003	0.007
	Scalar model	221.835 (109)	0.964	0.963	0.058 [0.047, 0.069]	42.469 (19)	0.002	−0.006	0.000

*df, degree of freedom; CFI, comparative fit index; TLI, Tucker–Lewis index; RMSEA, root mean square error of approximation; CI, confidence interval*.

The change-in-model fit indices across age group suggested that the fit of metric invariance model was slightly better relative to the configural invariance model (Δχ^2^ was non-significant, ΔCFI and ΔTLI were < 0.01, and ΔRMSEA was < 0.015), the metric invariance was supported. Similarly, the non-significant Δχ^2^ and small change in other indices of the scalar invariance model supported the scalar invariance. In terms of the employment group, metric invariance was established (Δχ2 was non-significant, ΔCFI and ΔTLI were < 0.01, and ΔRMSEA was < 0.015). Although the Δχ^2^ was significant (*p* = 0.002) of the scalar invariance model, the change of other fit indices indicated nuanced differences between the models. Therefore, the scalar invariance was established. Overall, the SDDS exhibited favorable measurement invariance across the sex, age, and employment group.

## Discussion

There are differences between the dependence of traditional drugs and synthetic drugs in terms of specific behaviors (Parrott, 2005; Tung et al., 2014; Engeli et al., 2020); however, the two also share similar characteristics. The dependence on traditional drugs and synthetic drugs is a cluster of cognitive, physical, and physiological symptoms (American Psychiatric, 2013) in which different factors may play different roles. In addition, the underlying physiological mechanism of these drugs is the dysregulation of the dopamine reward pathway, which would persist beyond detoxification (Koob and Le Moal, 2001). Therefore, it is not appropriate to adopt a one-size-fits-all approach or to confuse them with each other, thus seeking common ground while preserving their differences is a better choice and was the starting point of the present study. Our findings supported a method that adapted a traditional drug dependence scale for synthetic drugs, and the SDDS clearly displayed sound psychometric properties with a certain significance for synthetic drugs research and clinical treatment.

Deletion of item 5 (times of entering compulsory drug rehabilitation centers) is consistent with the study of Gu et al. (2008), indicating that the item was problematic. The item was designed as an objective indicator of failed detoxification by asking the times of entering compulsory drug rehabilitation centers, which is the primary drug rehabilitation modality in China and some Asian countries (Kamarulzaman and McBrayer, 2015); however, it ignored the effect of motivation. People in the compulsory detoxication center are those having severe drug dependence who refused to receive community-based rehabilitation or have failed to maintain abstinence (Yang et al., 2015), i.e., most of them have weaker incentives to cut down or control drug use. Furthermore, there are many external factors that affect the possibility of entering a compulsory drug rehabilitation center, such as the strictness of local drug supervision, tolerance of community residents, and the attitude of the family toward drugs (Zhang et al., 2016); therefore, the item ultimately could not reflect the desire or unsuccessful effort to decrease the drug use. This finding indicates the difficulty of quantifying the failed attempts to control the drug use, such that the number of entries into a voluntary detoxication center may be a better choice. Besides, the remaining items all exhibited good discrimination and internal consistency, meeting the psychometric standards of item quality.

The results of CFA supported the three-factor structure. Compared with the two-factor model, the three-factor model provides a more detailed classification and allows further exploration. Specifically, given that physical dependence and psychological dependence might play different roles in the mechanism of synthetic drug dependence (Degenhardt et al., 2010), it is then viable to compare the predictive effect of the two factors on, for example, drug craving and drug relapse, which is necessary to seek targeted treatment. Compared with the four-factor model, the physical damage and social function damage were combined into the functional impact factor, which indicated the close association of physical damage and social function damage. The items of physical damage factor assessed the change in health, sexual desire, and depressive feelings after taking drugs, the health of individual would affect the performance of daily activities (McAuley and Katula, 1998), severe health injury leads to the inability to work and study; decreased sexual desire would also have deleterious effects on the intimate relationship (Brezsnyak and Whisman, 2004); besides, the depressive mood would reduce the initiative and impact many aspects of social functions (Simon et al., 2007). Therefore, physical damage would influence the corresponding social functions reflected in the social function damage factor, and it is more appropriate to combine the two factors. As analyzed above, correlations between factors and validity variables exactly represented the specific role of each factor. Physical dependence was not significantly correlated with any relevant variables, suggesting that objective behavior indices, such as frequency and dosage, may represent the patterns of drug use (Bruno et al., 2009) directly affecting the physiological process, contributing to the neuro-adaption and incentive sensitization of drugs (Robinson and Berridge, 2001; Degenhardt et al., 2010), while having a weaker impact on personality change. Notably, psychological dependence was the only significant predictor in the regression analysis of criterion variables, suggesting that psychological factors play an important role in the development of drug dependence. In-line with the characteristic that physical symptoms of synthetic drugs are not as apparent as those of traditional drugs (Degenhardt et al., 2005; Wang et al., 2010), it can be posited that psychological factors might make a relatively greater contribution to synthetic drug dependence than to traditional drug dependence (Abdallah et al., 2007; Degenhardt et al., 2010).

More specifically, the psychological dependence factor in the current study includes, for example, attempts to detoxify and time spent on drugs, being similar to the impaired control dimension in the DSM diagnostic criteria, which is regarded as the key characteristic of the drug problem (Fillmore, 2003). Taking into account behavior theory, the inability to inhibit drug abuse and impulsive behavior are the core features of impaired control (Gullo et al., 2014). For individuals with impaired control ability, it would be difficult to regulate the dosage and frequency of drug taking even though they have the desire to control their drug use (Vaughan et al., 2019), eventually resulting in larger dosages and higher frequency. In addition, when exposed to aversive events, they would be more likely to take drugs because of the negative reinforcement role of drugs (O'Connor et al., 2009), increasing the possibility of relapse and making detoxification harder. Overall, the results of the present study illustrated the importance of impaired control in the formation and development of synthetic drug dependence, in which the cultivation and enhancement of control ability would make a difference to help synthetic drug abusers detoxify under clinical treatment. Unlike physical dependence and psychological dependence, acting as the demand for the drug, functional impact reflects the results of drug use, such as health conditions, changes in sexual desire, depression symptoms, and problems with the study, work, and social relationships, which is an effect, rather than a cause, thus they were related to the reward sensitivity, punishment sensitivity, and impulsivity, but did not have significant predictive power.

The SDDS revealed adequate measurement invariance across sex, age, and employment; however, one point is worth noting in the sex group. Item 2 did not have scalar invariance in the second threshold and third threshold, suggesting that female participants had a higher probability to respond from the second option to the third option and the third option to the fourth option. It may be related to the proportional distribution of the data, there were more balanced distributions of different options in males, while a larger proportion (64.6%) of the first two options (lower dosage of drug use) in females and caused a bigger difference between options than males. A possible explanation could be that women have a stronger episodic memory ability than men (Asperholm et al., 2019), thus women recall it more clearly, but men are prone to overestimate the dosage of drug use. For the age groups, the results supported the measurement invariance between young participants and middle-age participants, indicated that the scale structure was unaffected by age variation, while cautious interpretations should be made because the current study only divided people into 18–30-year old and 30-year old, a more detailed age division may demonstrate different results. The results of employment invariance signified the excellent measurement equivalence of the scale across employed people and unemployed people, suggesting that the SDDS is stable among people with or without occupation and thus can be applied to a wide range of participants. Besides, other economic factors, such as socioeconomic status (Gordon et al., 2020), personal income level, and family economic conditions (Trucco et al., 2014), can also impact substance use; therefore, future research examining the invariance of economic levels based on the current study could produce valuable results. In conclusion, the SDDS displayed good cross-population consistency and stability among men and women, middle age and young age groups, and employed and unemployed people; therefore, it can be used validly to assess and compare the dependence of synthetic drugs across different sex, age, and employment groups.

Despite the noteworthy advantages of the present study, its findings should be interpreted in light of several limitations. People in the drug rehabilitation center had received detoxification treatment in the environment in the absence of drug-related clues, thus their level of synthetic drug dependence would be reduced to some extent. To obtain more authentic drug dependence information, participants were required to recall their drug use behaviors, such as frequency, dosage, and health problems, before they entered the drug rehabilitation center; however, such a time interval would cause recall bias and weaken the reliability of the results. Besides, the SDDS is a self-rating scale, and subjects might be concerned that higher scores could incur punishment or other negative consequences, leading them to provide imprecise information. To address this problem, much work had been done to eliminate their worries *via* clear instructions, appropriate staff arrangements, and normative testing procedures. Thirdly, the current study focused on the compulsory detoxification population, which might differ in terms of detoxification motivation and self-control ability from a voluntary detoxification population; therefore, future research would benefit from including such a sample for further comparison and analysis. Lastly, the value of composite reliability of physical dependence factor was relatively low although it met the basic psychometric requirements, indicating that the associations between factors and items are not strong enough to obtain ideal exploratory power, which may be improved by changing the number of items or options in the future research (Bacon et al., 1995). Poor convergent validity may be rooted in the low reliability above, the choice of criterion variables was another possible explanation. Behavioral outcome variables which can reduce the instability of relations may be a better choice than the potential causal variables (Rohrer, 2018) such as the personality variables in the current study. For instance, drug-induced negative emotions (depression or anxiety), drug craving, and so on can be listed as the appropriate options.

The rapid increase in the prevalence of synthetic drugs and lack of specific measures require robust and short scales that measure the dependence on synthetic drugs in a wide range of people. The current research developed a psychometrically sound self-report measure, termed the SDDS. It consists of four subscales: physical dependence, psychological dependence, health injury, and social function injury. Good psychometric properties of the SDDS were gained in terms of the item quality, internal consistency, construct validity, convergent validity, and adequate measurement invariance across sex, age, and employment. Hence, the SDDS is an effective and valuable instrument to assess dependence on synthetic drugs and holds promise as a significant contributor to the clinical treatment of synthetic drugs abusers.

## Data Availability Statement

The datasets presented in this study can be found in online repositories. The names of the repository/repositories and accession number(s) can be found in the article/Supplementary Material.

## Ethics Statement

The studies involving human participants were reviewed and approved by Institutional Review Board of the Second Xiangya Hospital of Central South University. The patients/participants provided their written informed consent to participate in this study.

## Author Contributions

M-TL sorted data and wrote all the manuscript and files. JZ made a lot of efforts in sampling communication and arrangement. D-CZ and X-WL provided many suggestions on statistical analysis and design. Q-QC and P-WY helped collect the data. Z-LL provided many helpful suggestions and instructions during the interactive reviewing. T-SC designed the whole study and followed up all the procedures. All authors contributed to the article and approved the submitted version.

## Funding

This work was supported by the National Key Research and Development Program for Science and Technology under Grant (Number 2016YFC0800908).

## Conflict of Interest

The authors declare that the research was conducted in the absence of any commercial or financial relationships that could be construed as a potential conflict of interest.

## Publisher's Note

All claims expressed in this article are solely those of the authors and do not necessarily represent those of their affiliated organizations, or those of the publisher, the editors and the reviewers. Any product that may be evaluated in this article, or claim that may be made by its manufacturer, is not guaranteed or endorsed by the publisher.
