# Supplementary material for: Development and Psychometric Properties of the Synthetic Drug Dependence Scale in a Chinese Sample
Source: Front Psychol. 2021 Oct 26;12:717029. doi: 10.3389/fpsyg.2021.717029 (PMC8576389; doi:10.3389/fpsyg.2021.717029)
Supplement: Supplementary file 1 [file Table_1.DOCX]

Supplementary Material

**The Synthetic Drug Dependence Scale (SDDS)**

We are going to ask you some questions about your synthetic drug use **before entering the rehabilitation center**. For each question, please select one statement that most closely reflects your situation, and mark the answer with “√”.

Q1. How many times did you use synthetic drug per day?

(0) once a week or less (1) 2~5 times a week (2) 1 or 2 times/d (3) ≥3 times/d

Q2. What's the average amount of the drug you took each time (do not select the unused drug)?

Methamphetamine: (0) <15mg (1) 15~30mg (2) 31~60mg (3) >60mg

Ecstasy: (0) <75mg (1) 75~150mg (2) 151~200mg (3) >200mg

Ketamine: (0)<15mg (1) 15~30mg (2) 31~100mg (3) >100mg

Q3. As the duration of drug use increases, your desire for drugs becomes stronger?

(0) No (1) sometimes (2) often (3) always

Q4. The horizontal line below shows the degree of craving for synthetic drugs. The left end of the horizontal line shows no craving for drugs at all, and the right end shows strongest craving for drugs. According to your craving for drugs before entering the drug rehabilitation center, draw an "X" in the corresponding place on the line.

| 2  3  4  5  6  7  8  9  10  0  1  no crave |  |  |  |  |  |  |  |  |  |  | strong crave |
| --- | --- | --- | --- | --- | --- | --- | --- | --- | --- | --- | --- |

Q5. How many times have you received drug rehabilitation?

(0) once (1) 2 or 3 times (2) 4 or 5 times (3) ≥6 times

Q6. How much time did you spend on synthetic drugs use (including looking for drug, using drug, and thinking about drug, etc.) per day?

(0) almost no time (1) a small proportion of the time

(2) a large proportion of the time (3) almost all of the time

Q7. Compared with your health status before using drug, was your physical health getting worse?

(0) no change (1) worsened some (2) worsened much (3) worsened extremely much

Q8. Compared with your libido before drug use, were you experiencing reduced sex drive?

(0) no change (1) worsened some (2) worsened much (3) worsened extremely much

Q9. How often did you have depressive feeling (e.g., hard to cheer up, loss of interest in life and

entertainment)?

(0) no (1) sometimes (2) often (3) always

Q10. How did synthetic drug use affect your work (including doing house chores, business, etc.)?

(0)none (1) to some (less than half) extent

(2) to much (more than half) extent (3) to an extreme extent (unable to work or study)

Q11. How did synthetic drug use affect your relationship with your family or friends?

(0)none

(1) to some (less than half) extent

(2) to much (more than half) extent

(3) to an extreme extent (such as marriage breakdown, running away from home)

Q12. How did synthetic drug use affect your social activities (such as a wedding of friends)?

(0) none

(1) to some (less than half) extent

(2) to much (more than half) extent

(3) to an extreme extent (no interest in participating in any activities)

合成毒品成瘾量表

根据**入戒毒所前**使用合成毒品的状况，每道题选择一项最符合你情况的答案，在所选答案上划“√”。

1、你在入戒毒所前使用毒品的次数：（0）偶尔使用（如每周1次或更少）（1）不是每天必须使用（每周2-5次）（2）每天必须使用1-2次（3）每天必须使用3次或3次以上。

2、你在入戒毒所前每次吸食下列毒品的量（未使用的毒品不选）：

冰毒类**：**（0）<15mg （1）15~30mg （2）31~60mg （3）>60mg；

摇头丸：（0）<75mg （1）75~150mg （2）151~200mg （3）>200mg；

K 粉类：（0）<15mg （1）15~30mg （2）31~100mg （3）>100mg。

3、在入戒毒所前，随着吸毒持续时间的延长，你对毒品的渴望越来越强烈？

（0）没有 （1）偶尔 （2）有时 （3）经常

4、下面这条横线从左到右表示对毒品渴求程度由轻到重，横线最左端表示对毒品无任何渴求，最右端表示对毒品强烈渴求，根据你入戒毒所前对毒品的渴求程度，在横线上相应的地方划“X”。

| 无渴求  2  3  4  5  6  7  8  9  10  0  1 |  |  |  |  |  |  |  |  |  |  | 强烈渴求 |
| --- | --- | --- | --- | --- | --- | --- | --- | --- | --- | --- | --- |

5、你到正规戒毒机构（强戒所或劳教所）戒过几次毒？

（0）第一次 （1）2-3次 （2）4-5次 （3）6次以上

6、你在一天中花在毒品上的时间（包括想毒、找毒、吸毒）：

（0）没有特别考虑过（1）少部分时间 （2）大部分时间 （3）整天想毒、找毒、吸毒。

7、同吸毒前比较，吸毒后你的健康状况：

（0）无变化 （1）差一些 （2）差多了 （3）非常差。

8、同吸毒前比较，吸毒后你的性生活及性欲变化：（0）无变化（1）性欲减弱，性生活次数略有减少 （2）性欲明显减弱，性生活次数明显减少 （3）性欲消失，无性生活。

9、吸毒后你是否觉得生活没意思，高兴不起来，对工作、娱乐、生活兴趣下降了？

（0）没有 （1）偶尔 （2）有时 （3）经常

10、吸毒对你工作或学习（包括经商、做家务等）的影响：（0）无影响（1）受到很少影响（2）受到一些影响，但尚能坚持工作或学习（3）受到有极大影响，不能工作或学习。

11、你是否因为吸毒同家人或朋友发生过矛盾？

（0）从未有过任何矛盾 （1）偶尔发生矛盾 （2）经常发生矛盾

（3）发生严重矛盾（如婚姻破裂、本人离家出走、与家庭断绝关系）

12、是否因为吸毒影响了你参加重要的社交活动（如亲戚朋友的婚礼）？ （0）不影响，都参加 （1）大部分都参加 （2）大部分都不参加 （3）对任何活动都没有兴趣参加
